# The impact of C-reactive protein testing on treatment-seeking behavior and patients’ attitudes toward their care in Myanmar and Thailand

**DOI:** 10.4081/hls.2023.11278

**Published:** 2023-07-06

**Authors:** Rachel C. Greer, Thomas Althaus, Sabine Dittrich, Christopher C. Butler, Phaik Yeong Cheah, Tri Wangrangsimakul, Frank M. Smithuis, Nicolas P.J. Day, Yoel Lubell

**Affiliations:** 1 Mahidol Oxford Tropical Medicine Research Unit, Faculty of Tropical Medicine, Mahidol University, Bangkok, Thailand; 2 Centre for Tropical Medicine and Global Health, Nuffield Department of Medicine, University of Oxford, Oxford, UK; 3 The Department of Health Action, Monaco, Monaco; 4 Monaco Scientific Centre, Monaco, Monaco; 5 FIND, global alliance for diagnostic, Geneva, Switzerland; 6 Deggendorf Institute of Technology, European-Campus Rottal Inn, Pfarrkirchen, Germany; 7 Clinical Trials Unit, Nuffield Department of Primary Care Health Sciences, University of Oxford, Oxford, UK; 8 The Ethox Centre, Nuffield Department of Population Health, University of Oxford, Oxford, UK; 9 Myanmar Oxford Clinical Research Unit, Yangon, Myanmar; 10 Medical Action Myanmar, Yangon, Myanmar

**Keywords:** C reactive protein, antibiotics, AMR, point of care testing, healthcare-seeking behaviour

## Abstract

C-reactive protein (CRP) point-of-care testing can reduce antibiotic prescribing in primary care patients with febrile and respiratory illness, yet little is known about its effects on treatment-seeking behavior. If patients go on to source antibiotics elsewhere, the impact of CRP testing will be limited. A randomized controlled trial assessed the impact of CRP testing on antibiotic prescriptions in Myanmar and Thai primary care patients with a febrile illness. Here we report patients’ treatment-seeking behavior before and during the two-week study period. Self-reported antibiotic use is compared against urine antibacterial activity. Patients’ opinions towards CRP testing were evaluated. Antibiotic use before study enrolment was reported by 5.4% while antimicrobial activity was detected in 20.8% of samples tested. During the study period, 14.8% of the patients sought additional healthcare, and 4.3% sourced their own antibiotics. Neither were affected by CRP testing. Overall, patients’ satisfaction with their care and CRP testing was high. CRP testing did not affect patients’ treatment-seeking behavior during the study period whilst modestly reducing antibiotic prescriptions. CRP testing appears to be acceptable to patients and their caregivers.

## Introduction

C-reactive protein (CRP) point of care (POC) testing can improve antibiotic prescribing by reducing initial antibiotic prescriptions for adults and children attending primary care with respiratory tract infections (RTIs).^1,2^ The majority of RTIs are viral and do not benefit from antibiotics, but despite this, RTIs remain a common reason for an antibiotic prescription. High levels of antibiotic prescribing have been reported in Southeast Asia; situational analyses of public primary care facilities in Myanmar revealed that antibiotics were prescribed to 87% (range 73-96%) of patients with upper respiratory tract infections (URTIs), while in Thailand, 43% (20-52%) were prescribed antibiotics during 2014 and 2015.^3^ Thailand has been active in developing antimicrobial stewardship policies and plans, and this appears to be reducing antibiotic prescriptions for URTIs.^3–5^ Optimal use of antibiotics is key to reducing the burden of antimicrobial resistance. In 2019, an estimated 254,000 deaths were attributable to bacterial resistance in Southeast Asia.^6^

CRP is an acute-phase protein that is raised in infection and inflammation. It can be measured at the POC using a finger prick blood test. Qualitative studies suggest that the majority of patients view the CRP POC test favorably.^7–9^ Less is known about its effect on treatment-seeking behavior after the initial consultation and whether patients comply with the recommendation to take or more likely *not* to take antibiotics. Researchers and healthcare workers have suggested that patients will go on to seek healthcare or antibiotics from other sources if they are unsatisfied with consultations using CRP POC tests.^8^ Whilst in research settings consultation at study sites has been largely unaffected by CRP interventions, there is a paucity of data on CRP testing’s effect on seeking healthcare and antibiotics from alternative sources.^4,10–14^ Trial participants report conflicting views about CRP testing’s impact on future care-seeking; some report that they will re-attend to receive another test (medicalizing a self-limiting illness) while others will delay seeking care as antibiotics were not needed.^7,12^ Patient-reported antibiotic use can be difficult to assess and validate due to a lack of awareness or understanding of antibiotics and other medications being taken, as well as poor adherence to treatment and recall times. Measuring urine antibacterial activity is one way to verify whether antibiotics are being taken currently.^10,15–17^ We conducted a randomized controlled trial (RCT) to evaluate CRP-guided antibiotic prescribing for patients attending primary care with an acute febrile illness. The primary outcomes have been reported previously.^4^ In summary, a modest reduction (39% *vs*. 34%) in antibiotic prescribing was seen in the intervention arm using a CRP cut-off of 40mg/L compared with the control arm (aOR 0.80, 95% CI 0.65-0.98). Patients with a high CRP level were more likely to receive an antibiotic and those with a low CRP were less likely to receive an antibiotic in the intervention arms compared to the control arm. Clinical outcomes were not affected.^4^

In this paper, we describe the secondary outcomes of patients’ treatment-seeking behavior (healthcare and antibiotics) before and during the two-week study period and compare self-reported antibiotic use against urine antibacterial activity. We explore patients’ and their caregivers’ views toward CRP POC testing.

## Materials and Methods

We conducted a multicentre, open–label RCT in Myanmar and Thailand. The trial design details have been reported previously.^4^ In brief, we recruited patients aged 1 year or older attending primary care with a documented fever (>37.5°C) or history of fever in the last 2 weeks. Patients were individually randomized 1:1:1 into intervention arm A (CRP cut-off of 20mg/L), intervention arm B (CRP cut-off of 40mg/L), or the control arm (standard care). These CRP cut-offs were based on reported CRP levels in Southeast Asian febrile patients and recent RCTs on POC CRP testing in primary care. Prior antibiotic use did not prevent participation.^4^

Healthcare workers were advised that patients with a low CRP result (defined by the intervention arm’s threshold) were unlikely to benefit from antibiotics while those with a high CRP were more likely to benefit from antibiotics. All patients were followed up on days 5 and 14. Urine samples were collected on day 0 and day 5. Opinions towards their care and CRP testing were ascertained by the researcher using close-ended questions on day 14.

### Study sites

The study sites in Myanmar included three not-for-profit clinics which provide primary healthcare for marginalized people and one government outpatient department. All patients were treated for free by doctors. The Thai study sites were six government-run primary care units that provide universal health care and medication to Thai citizens for a nominal fee. They are usually staffed by nurses and public health officers. In both Myanmar and Thailand, antibiotics are also available from multiple sources, such as pharmacies and village shops without a prescription.

### Laboratory procedures

Urine antibacterial activity was tested at the Mahidol Oxford Tropical Medicine Research Unit (MORU) laboratory in Bangkok, Thailand. The reference organism, *Bacillus stearothermophilus* (ATCC 7953) was plated on Mueller Hinton agar. Urine samples were thawed and then 3 μL samples were pipetted onto a blank filter paper noting the disc position. Plates were incubated aerobically at 56°C for 18 to 24 hours. If an inhibitory zone was seen around the urine sample then antibacterial activity was declared.^16^ Samples were tested in duplicate and divergent results were repeated. All urine samples collected on day 5 were tested for antibacterial activity but only a subset of day 0 urine samples (409/2,292, 17.8%) were tested due to resource constraints.

CRP levels were assessed using the NycoCard II Reader, Axis-Shield, Oslo, Norway. Capillary blood samples were tested at point-of-care for intervention patients whereas for control patients venous samples were retrospectively tested in MORU’s local laboratories.^18^

### Statistical analysis

Categorical data were summarised using counts and percentages, and compared using χ^2^ tests. Mann-Whitney U tests were used to compare scores without normal distribution. Logistic regression models were used to evaluate indicators of treatment-seeking behavior during the study, with the study sites fitted as random effects. Univariate analyses of the potential indicators of treatment-seeking behavior were performed and significant variables (p<0.05) were added to multivariable analyses. Agreement between patient-reported antibiotic use and urine antibacterial activity was assessed using the kappa statistic. Patients’ consultation experience scores were created using the sum of responses to questions 2, 3, 4, 8, and 9 (Table 1). Responses were recoded so that positive answers received 1 point, neutral answers 0 points, and negative responses -1 point.^18^

## Results

### Health-seeking and antibiotic use before enrolment

The RCT enrolled 2,410 patients with an acute fever or history of fever presenting to primary care in Myanmar and Thailand between 2016 and 2017.^4^ Over half of the patients (1,372/2,408, 57%) had sought healthcare in the two weeks before study enrolment, most frequently from pharmacies (53.8%) and clinics (22.1%). Prior care was more likely to have been sought by patients in the Myanmar facilities, as compared with those in the Thai facilities (74.9% *vs* 38.4%, p<0.001), and when the patient was an adult as compared with children (61.5% *vs* 52.4%, p<0.001).

New medication had been taken by 1,732/2,409 (71.9%) of the patients in the 2 weeks before study enrolment; of these, 367 (21.2%) had taken at least one unknown medication. Antibiotics had been knowingly taken by 130/2,409 (5.4%). Sources of antibiotics include clinics (81/126, 62.3%), pharmacies (30, 23.1%), hospitals (6, 4.6%), natural healers (5, 3.9%), household supplies (3, 2.3%), street vendors (1, 0.8%) and unknown (4, 3.1%). A minority of those who had sought healthcare reported taking antibiotics (127/1,372, 9.3%). Prior antibiotic use did not vary between Myanmar and Thai patients or adults and children (p=0.347 and 0.223, respectively).

**Figure 1:**
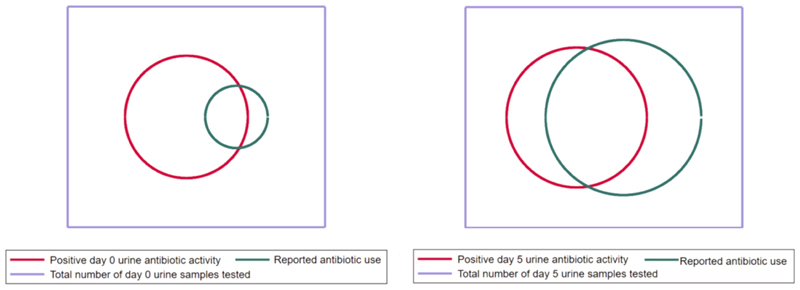
Venn diagrams to show day 0 and day 5 urine antibacterial activity and reported antibiotic use. Adapted from Greer 2022.^18^

Antibacterial activity was found in 85/409 (20.8%) of the urine samples tested at enrolment. The agreement between reported antibiotic use and urine antibacterial activity was 81.2% (kappa = 0.21). Of the 409 patients, 22 reported antibiotic use in the 48 hours before the test, of whom 15 were positive and 7 were negative for antibacterial activity, while 70/85 (82.4%) of the patients with urine antibacterial activity did not report antibiotic use (Figure 1). In those who were taking an unknown medication, 29/61 (47.5%) of the urine samples were positive for antibacterial activity.^18^

### Health-seeking and antibiotic use after enrolment

Antibiotics were prescribed at enrolment to 515/1,593 (32.3%) of the patients in the CRP intervention arms compared to 297/799 (37.2%, p=0.018) in the control arm. This reduction in prescribing was primarily due to a reduction in Myanmar adults.^4^ During the study period, healthcare was sought by 339/2,294 (14.8%) of the patients (from any source or facility other than the study follow-up visits). There was no difference between those in the CRP intervention arms and the control arm (p=0.552, Supplementary Table 1). In the multivariable analysis, significantly less care was sought during the study by Thai patients and those who had received an antibiotic at enrolment. Significantly more care was sought by those who had sought care before the study, presented with a documented fever, higher self-reported symptom severity, higher CRP results, and those diagnosed with an unspecified acute viral or dual infection compared to those with RTIs (Table 2).

Antibiotics were prescribed to 110/2,311 (4.8%) of the patients on day 5 and 15/2,317 (0.7%) on day 14. In addition, 95/2,206 (4.3%) of the patients sourced their antibiotics, approximately a third of the 254 patients seeking care elsewhere; an additional 79 patients received an unknown medication. There was no difference between those seeking antibiotics in the CRP intervention or control arms. The only significant variable in the univariate analyses for seeking antibiotics during the study was having a higher CRP result at enrolment, p=0.002 (Supplementary Table 1).

On day 5, urine antibacterial activity was found in 521/2,065 (25.2%) of the samples (Figure 1). The overall agreement between patient-reported antibiotic use and urine antibacterial activity was 77.4% (kappa=0.46). In the preceding 48 hours, 641/2,065 (31.0%) patients reported antibiotic use; 352 (54.9%) samples were positive and 289 (45.1%) were negative. In those with urine antibacterial activity, 352/521 (67.6%) patients had reported antibiotic use, while 155 (29.8%) reported no antibiotic use. Most (77.6%) patients reported knowing whether they had been prescribed an antibiotic at enrolment; the rest were unsure when asked on day 14. Adherence to antibiotic courses was reported by 687/829 (86.7%) of the patients.^18^

### Patients’ and caregivers’ opinions and attitudes toward the consultation and CRP testing

On day 14, all patients were asked about their care, and those in the intervention arms were asked additional questions about CRP testing. Half of the patients answered these questions themselves while the other half were answered by their parents or guardians. Overall satisfaction with the care received was very high (Table 1). There were no differences between the intervention and control arms in terms of consultation scores (p=0.980), an adequate explanation of the treatment (p=0.966), or agreement with the treatment (p=0.864). Thai patients rated each of these higher than Myanmar patients (p<0.001).

Patients who sought further healthcare during the study scored lower for their consultation experience (p<0.001), an adequate explanation of the treatment (p=0.007), and agreement with their treatment (p=0.006) than those who did not. Patients who sourced antibiotics during the study had similar consultation scores (p=0.313) and an adequate explanation of the treatment (p=0.847) but reported less agreement with treatment (p=0.001). Agreement with the treatment was also lower in those not prescribed an antibiotic at enrolment compared to those who were (p=0.033), however over 80% agreed with the antibiotic prescribing decision.

In total, 67.3% of the patients reported receiving enough explanation to understand their treatment. While in the intervention arms, 61.1% felt the objective of the CRP test was clear and 56.6% reported that the test results were explained in a way that they understood. The majority of intervention patients wanted the CRP test to be used again, felt more confident whether antibiotics were needed, and that it improved their quality of care.^18^

## Discussion

Following a modest reduction in prescribing after the first presentation, CRP POC testing did not affect patients’ treatment-seeking behavior during the two-week study period. Patients expressed positive opinions towards CRP testing and its use in future consultations. Studies from Asia and Europe have reported no difference in re-attendances between patients in CRP and control arms.^4,10–14,19^ Our study goes further by showing no difference in the numbers of patients seeking additional healthcare or antibiotics in the two weeks following first attendance at the study facility. It is encouraging that despite the relatively low antibiotic prescribing in the control and intervention arms, less than 5% of the patients went on to source their antibiotics. Taken together with the reported high adherence to antibiotic courses this should encourage healthcare workers and policymakers that most patients will comply with antibiotic treatment plans, even when antibiotics can be sought from other sources. Patients reported high levels of satisfaction with their care. This is consistent with other studies on CRP POC testing.^10–12^

Our study raises concerns about unknown medication use. A fifth of those taking a new medication before enrolment did not know what they were taking. Even within the context of a trial focusing on antibiotic use a quarter did not know if they were prescribed an antibiotic as part of the study. Some of this uncertainty about antibiotic use may be explained by the multiple terms used for antibiotics in Thailand and the lack of a formal word for antibiotics in Myanmar.^20,21^ This uncertainty is likely to be reflected in the differences between reported antibiotic use and urine antibacterial activity and is consistent with other studies that found lower levels of reported antibiotic use compared to urine antibacterial activity.^15,16,22^ Another reason for this discrepancy may be environmental exposure to antibiotics, foods or chemicals with antibacterial activity.^16,23^ False-negative results may have been caused by non-adherence to antibiotics, extra-renal antibiotic excretion, reduced test sensitivity due to one reference organism being used, and the freezing and thawing of urine samples.^15–18,22^ Moving forward patients need to be aware of their antibiotic use if they are to be involved in strategies to optimize antibiotic use.

Patients’ understanding of the CRP test could be improved and may help to increase the impact of CRP testing and patients’ agreement with their antibiotic treatment. The patients who did seek additional healthcare during the study had lower consultation experience scores, less adequate explanation, and less agreement with their treatment. Further work is required to explore how these areas could be addressed in future interventions. Special focus needs to be given to patients who are not prescribed an antibiotic, especially when antibiotics are expected and this is the prescribing norm.

This manuscript adds detailed treatment-seeking behavior to the results of our CRP POC RCT. Combined with the urine antibacterial activity data and patient’s opinions towards CRP POC testing this provides a more holistic review of the patient’s acceptance of CRP POC testing, in the context of two low-and-middle-income countries. There are, however, several limitations to our study; the effect of CRP testing on treatment-seeking behavior may have differed if the intervention had had a larger impact on antibiotic prescribing. Patients’ satisfaction with their care may have been influenced by the study design, including the follow-up visits. Opinions towards CRP testing were assessed using close-ended questions which cannot give as detailed or nuanced answers as qualitative methods. However, as part of our wider work patients’ views were explored using semi-structured interviews and their opinions were widely positive.^8^ Due to resource constraints we were unable to test all the enrolment urine samples for antibacterial activity and were only able to use one reference organism; this may have led to an underestimation of urine antibacterial activity. Our study sites were limited to government and not-for-profit-run primary care clinics so the results may not be generalizable to other facilities.

## Conclusions

The use of CRP POC testing has been shown to improve healthcare workers’ antibiotic prescribing practices. Here it was shown that CRP testing was widely acceptable to primary care patients in Myanmar and Thailand, without affecting subsequent treatment-seeking behavior. Encouragingly, the vast majority of antibiotics obtained outside the study facilities came from formal sources, such as pharmacies and clinics, even though in Myanmar and Thailand antibiotics are widely available from informal providers, such as natural healers or street vendors. These formal providers could be easier sites to target antimicrobial stewardship interventions. Healthcare workers should communicate to patients when they are prescribing antibiotics and if they are not the reasons why antibiotics are not required.

## Supplementary Material

Supplementary materials

## Figures and Tables

**Table 1:** Patients’ and caregivers’ opinions and attitudes towards the consultation and CRP POC testing, by country and intervention. Adapted from Greer 2022.^18^

Patients’ and caregivers’ opinions & attitudes	Agree n (%)	Neutral n (%)	Disagree n (%)
**I think that the healthcare worker’s decision to prescribe or not to prescribe an antibiotic for my treatment was correct (Q 2)**			
Intervention arms (N = 1,377)	1,113 (80.8)	241 (17.5)	23 (1.7)
Control arm (N = 691)	556 (80.5)	125 (18.1)	10 (1.5)
Thailand (N = 1,172)	1,107 (94.5)	49 (4.2)	16 (1.4)
Myanmar (N = 896)	562 (62.7)	317 (35.4)	17 (1.9)
**I did not get enough explanation to understand the treatment (Q 3)**			
Intervention arms (N = 1,448)	79 (5.5)	394 (27.2)	975 (67.3)
Control arm (N = 725)	37 (5.1)	200 (27.6)	488 (67.3)
Thailand (N = 1,173)	54 (4.6)	269 (22.9)	850 (72.5)
Myanmar (N = 1,000)	62 (6.2)	325 (32.5)	613 (61.3)
**I felt that the consultation was too fast (Q 4)**			
Intervention arms (N = 1,451)	335 (23.1)	254 (17.5)	862 (59.4)
Control arm (N = 726)	155 (21.4)	123 (16.9)	448 (61.7)
Thailand (N = 1,174)	394 (33.6)	36 (3.1)	744 (63.4)
Myanmar (N =1,003)	96 (9.6)	341 (34.0)	566 (56.4)
**I fully understood the instructions for taking the prescribed antibiotic (including when, how much, how often, and how long I have to take the medication) (Q 5)**			
Intervention arms (N = 407)	388 (95.3)	15 (3.7)	4 (1.0)
Control arm (N = 211)	195 (92.4)	14 (6.6)	2 (1.0)
Thailand (N = 353)	343 (97.2)	8 (2.3)	2 (0.6)
Myanmar (N = 265)	240 (90.6)	21 (7.9)	4 (1.5)
**It is too much effort to come to the health center for the treatment that I received (Q 8)**			
Intervention arms (N = 1,461)	107 (7.3)	212 (14.5)	1,142 (78.2)
Control arm (N = 732)	58 (7.9)	101 (13.8)	573 (78.3)
Thailand (N = 1,173)	32 (2.7)	30 (2.6)	1,111 (94.7)
Myanmar (N = 1,020)	133 (13.0)	283 (27.8)	604 (59.2)
**Overall, I am satisfied with my care (Q 9)**			
Intervention arms (N = 1,464)	1,429 (97.6)	33 (2.3)	2 (0.1)
Control arm (N = 730)	709 (97.1)	19 (2.6)	2 (0.3)
Thailand (N = 1,173)	1,155 (98.5)	16 (1.4)	2 (0.2)
Myanmar (N = 1,021)	983 (96.3)	36 (3.5)	2 (0.2)
**Intervention arms only**			
**The objective of the finger-prick CRP test is not clear to me (Q 6)**			
All (N = 1,453)	64 (4.4)	502 (34.6)	887 (61.1)
Thailand (N = 776)	31 (4.0)	292 (37.6)	453 (58.4)
Myanmar (N = 677)	33 (4.9)	210 (31.0)	434 (64.1)
**The finger-prick test for CRP is painless (Q 7)**			
All (N = 1,450)	998 (68.8)	222 (15.3)	230 (15.9)
Thailand (N = 777)	672 (86.5)	36 (4.6)	69 (8.9)
Myanmar (N = 673)	326 (48.4)	186 (27.6)	161 (23.9)
	**Yes**	**Do not know**	**No**
**Did the health worker explain the finger-prick test results to you in a way that you understood? (Q 10**)			
All (N = 1,450)	821 (56.6)	299 (20.6)	330 (22.8)
Thailand (N = 774)	435 (56.2)	194 (25.1)	145 (18.7)
Myanmar (N = 676)	386 (57.1)	105 (15.5)	185 (27.4)
**Would you like the health worker to use the finger-prick test for CRP again the next time you have an illness? (Q 14)**			
All (N = 1,461)	1,329 (91.0)	103 (7.1)	29 (2.0)
Thailand (N = 778)	763 (98.1)	12 (1.5)	3 (0.4)
Myanmar (N = 683) **Did the health worker seem to base his/her treatment decision on the test results? (Q 12)**	566 (82.9)	91 (13.3)	26 (3.8)
All (N = 1,443)	782 (54.2)	557 (38.6)	104 (7.2)
Thailand (N = 774)	492 (63.6)	273 (35.3)	9 (1.2)
Myanmar (N = 669)	290 (43.4)	284 (42.5)	95 (14.2)
**Patients’ and caregivers’ opinions & attitudes**	**Too much**	**Enough/adequately**	**Not enough**
**If so: Do you think the health worker relied too much, enough, or not enough on the test results when he/she made the treatment decision? (Q 12a)**			
All (N = 778)	192 (24.7)	580 (74.6)	6 (0.8)
Thailand (N = 491)	181 (36.9)	309 (62.9)	1 (0.2)
Myanmar (N = 287)	11 (3.8)	271 (94.4)	5 (1.7)
	**More confident**	**Neither more nor less confident**	**Less confident**
**Did the finger-prick test make you feel more or less confident that antibiotics are needed / not needed for your illness? (Q 11)**			
All (N = 1,432)	1,201 (83.9)	225 (15.7)	6 (0.4)
Thailand (N = 776)	738 (95.1)	37 (4.8)	1 (0.1)
Myanmar (N =656)	463 (70.6)	188 (28.7)	5 (0.8)
	**Improves**	**No difference, unsure**	**Worsens**
**Do you feel that the finger-prick test for CRP improves or worsens the quality of the care you receive? (Q 13)**			
All (N = 1,446)	1,281 (88.6)	165 (11.4)	0
Thailand (N = 778)	753 (96.8)	25 (3.2)	0
Myanmar (N = 668)	528 (79.0)	140 (21.0)	0

**Table 2:** Multivariable logistic regression of variables associated with seeking healthcare during the RCT. Adapted from Greer 2022.^18^

Variable	Additional healthcare sought during the study period	
	aOR* (95% CI)	P value
Country		
Myanmar patients	Reference	
Thai patients	0.43 (0.23 to 0.81)	0.008
Sought healthcare before enrolment	1.47 (1.07 to 2.01)	0.016
Documented fever at enrolment	1.75 (1.31 to 2.35)	<0.001
Self-reported symptom severity score (1 point increase)	1.81 (1.33 to 2.46)	<0.001
Diagnosis at enrolment#		
RTIs	Reference	
Other infections	1.22 (0.70 to 2.12)	0.480
Acute viral infections (unspecified)	1.71 (1.12 to 2.63)	0.014
Dual infection	1.82 (1.04 to 3.18)	0.037
CRP level at enrolment (1 mg/L increase)	1.01 (1.00 to 1.01)	0.001
Antibiotics prescribed at enrolment	0.52 (0.37 to 0.73)	<0.001

* The study site was added as a random effect. #Other infections include all non-RTIs affecting other systems such as gastrointestinal and skin infections. Acute viral infection was a common diagnosis made in Myanmar alongside RTIs, common symptoms included cough and runny nose but some patients had fever as the sole symptom. Dual infections include a diagnosis from two of the diagnosis categories.

## Data Availability

data access will be granted upon reasonable request from the Mahidol Oxford Tropical Medicine Research Unit’s Data Access Committee. Instructions and the data application form are available from: https://www.tropmedres.ac/units/moru-bangkok/bioethics-engagement/data-sharing.
