# Supplementary material for: The impact of C-reactive protein testing on treatment-seeking behavior and patients’ attitudes toward their care in Myanmar and Thailand
Source: Healthc Low Resour Settings. Author manuscript; Available in PMC 2024 Feb 8. (PMC7615608; doi:10.4081/hls.2023.11278)
Supplement: Supplementary materials [file EMS181538-supplement-Supplementary_materials.pdf]

Supplementary Materials

Table S1. Univariate analyses of variables for seeking healthcare and antibiotics during the RCT study period. Adapted from Greer 2022.[18]

**Variable**	**Additional healthcare during the study**		**Additional antibiotics during the study**	
	**aOR* (95% CI)**	**P value**	**aOR* (95% CI)**	**P value**
Randomization arm				
• Control arm	Reference			
• CRP intervention arms	1.08 (0.84 to 1.39)	0.552	1.09 (0.70 to 1.70)	0.708
Country				
• Myanmar	Reference			
• Thailand	0.38 (0.22 to 0.66)	0.001	0.76 (0.33 to 1.71)	0.502
Sex				
• Female	Reference			
• Male	1.17 (0.93 to 1.48)	0.185	1.08 (0.71 to 1.63)	0.729
Age				
• Adults and adolescents	Reference			
• Children	0.86 (0.68 to 1.09)	0.215	0.80 (0.52 to 1.22)	0.294
Higher than primary education†	1.03 (0.79 to 1.34)	0.805	1.21 (0.76 to 1.91)	0.420
Profession†				
• Agriculture	Reference			
• Non-skilled laborer	1.03 (0.68 to 1.56)	0.895	0.82 (0.45 to 1.49)	0.522

• Skilled laborer or professional	1.12 (0.71 to 1.76)	0.630	0.91 (0.47 to 1.77)	0.474
• No employment	1.21 (0.70 to 2.08)	0.504	0.70 (0.27 to 1.85)	0.777
Sought healthcare before enrolment	1.58 (1.20 to 2.09)	0.001	1.51 (0.94 to 2.40)	0.087
Antibiotics before enrolment	1.35 (0.84 to 2.18)	0.217	0.87 (0.34 to 2.21)	0.771
Travel > 30 minutes to the study site	0.79 (0.59 to 1.08)	0.138	1.06 (0.62 to 1.81)	0.820
Self-declared chronic disease	1.35 (0.99 to 1.84)	0.057	0.93 (0.51 to 1.70)	0.812
Duration of symptoms (1-day increase)	1.02 (0.97 to 1.07)	0.477	1.08 (0.98 to 1.18)	0.113
Self-reported symptom severity (1 point increase)	1.72 (1.29 to 2.29)	< 0.001	1.26 (0.83 to 1.92)	0.282
Documented fever > 37.5°C at enrolment	1.62 (1.27 to 2.07)	< 0.001	1.57 (1.02 to 2.41)	0.038
CRP level at enrolment (1 mg/L increase)	1.01 (1.00 to 1.01)	0.001	1.01 (1.00 to 1.01)	0.002
Abnormal examination at enrolment•	0.89 (0.67 to 1.19)	0.426	0.89 (0.55 to 1.45)	0.646
Diagnosis at enrolment[#]				
• RTIs	Reference			
• Other infections	1.30 (0.79 to 2.14)	0.304	0.75 (0.26 to 2.14)	0.586
• Acute viral infections (unspecified)	1.88 (1.28 to 2.75)	0.001	1.20 (0.58 to 2.51)	0.623
• Dual infection	1.72 (1.01 to 2.94)	0.045	0.68 (0.20 to 2.25)	0.525
Antibiotics prescribed at enrolment	0.71 (0.55 to 0.93)	0.012	0.75 (0.47 to 1.20)	0.227

Antibiotic prescription in concordance with CRP result	0.82 (0.58 to 1.16)	0.255	1.10 (0.59 to 2.06)	0.760

*The study site was added as a random effect. †The education level and profession are for the participant unless they were under the age of 18, in which case the head of the household is used. •Abnormal examination finding: excludes observations. #Other infections include all non-RTIs such as gastrointestinal and skin infections. Acute viral infection was a common diagnosis made in Myanmar alongside RTIs, common symptoms included cough and runny nose but some patients had fever as the sole symptom. Dual infections include a diagnosis from two of the diagnosis categories
